# Influence of Fe and Cu Co-Doping on Structural, Magnetic and Electrochemical Properties of CeO_2_ Nanoparticles

**DOI:** 10.3390/ma15124119

**Published:** 2022-06-09

**Authors:** Shalendra Kumar, Faheem Ahmed, Naushad Ahmad, Nagih M. Shaalan, Rajesh Kumar, Adil Alshoaibi, Nishat Arshi, Saurabh Dalela, Parvez Ahmad Alvi, Kavita Kumari

**Affiliations:** 1 Department of Physics, College of Science, King Faisal University, P.O. Box 400, Al-Ahsa 31982, Saudi Arabia; fahmed@kfu.edu.sa (F.A.); nmohammed@kfu.edu.sa (N.M.S.); adshoaibi@kfu.edu.sa (A.A.); 2 Department of Physics, University of Petroleum & Energy Studies, Dehradun 248007, India; 3 Department of Chemistry, College of Science, King Saud University, P.O. Box 2455, Riyadh 11451, Saudi Arabia; anaushad@ksu.edu.sa; 4 Physics Department, Faculty of Science, Assiut University, Assiut 71516, Egypt; 5 University School of Basic and Applied Sciences, Guru Gobind Singh Indraprastha University, New Delhi 110078, India; rajeshpositron@gmail.com; 6 Department of Basic Sciences, Preparatory Year Deanship, King Faisal University, P.O. Box 400, Al-Ahsa 31982, Saudi Arabia; nshastri@kfu.edu.sa; 7 Department of Pure & Applied Physics, University of Kota, Kota 324005, India; sdphysics@rediffmail.com; 8 Department of Physics, Banasthali Vidyapith, Banasthali 304022, India; drpaalvi@gmail.com; 9 School of Materials Science and Engineering, Changwon National University, Changwon 51140, Gyeongnam, Korea; kkmalhan@gmail.com

**Keywords:** CeO_2_, XRD, magnetic properties, dc magnetization, DMS, supercapacitor, electrochemical properties

## Abstract

The nanoparticles of CeO_2_, Ce_0.98_Fe_0.02_O_2_, and Ce_0.78_Fe_0.02_Cu_0.20_O_2_ were synthesized using the co-precipitation-synthesis technique. The effect of co-doping of Fe and Cu on structural, optical, and magnetic properties as well as specific capacitance have been studied using X-ray diffraction (XRD), scanning-electron microscopy (SEM), UV-visible spectroscopy, Raman spectroscopy, dc magnetization, and electrochemical measurements at room temperature. The results of the XRD analysis infer that all the samples have a single-phase nature and exclude the formation of any extra phase. Particle size has been found to reduce as a result of doping and co-doping. The smallest particle size was obtained to be 5.59 nm for Ce_0.78_Fe_0.02_Cu_0.20_O_2_. The particles show a spherical-shape morphology. Raman active modes, corresponding to CeO_2_, were observed in the Raman spectra, with noticeable shifting with doping and co-doping indicating the presence of defect states. The bandgap, calculated using UV-Vis spectroscopy, showed relatively low bandgap energy (1.7 eV). The dc magnetization results indicate the enhancement of the magnetic moment in the samples, with doping and co-doping. The highest value of saturation magnetization (1.3 × 10^−2^ emu/g) has been found for Ce_0.78_Fe_0.02_Cu_0.20_O_2_ nanoparticles. The electrochemical behavior studied using cyclic-voltammetry (CV) measurements showed that the Ce_0.98_Fe_0.02_O_2_ electrode exhibits superior-specific capacitance (~532 F g^−1^) along with capacitance retention of ~94% for 1000 cycles.

## 1. Introduction

Metal-oxide nanostructures have attracted the attention of the scientific community due to their enormous potential technological applications [1,2,3,4]. Among these nanomaterials, cerium-oxide (CeO_2_) nanostructures have appealed to researchers due to a wide range of applications, such as UV filters, UV blockers, energy-storage applications, spintronics, magneto-optoelectronics, glass-polishing materials, photocatalytic behavior to degrade dyes, automobile-exhaust promoters, and solid-state fuel cells (SOFC) [5,6,7,8,9,10,11,12,13,14]. During the last few decades, the spintronics (spin-transport electronics or spin-based electronics), also known as magneto electronics, have been widely studied to develop technological applications that utilize both the magnetic moment associated with an electron spin and their fundamental electronic charge in solid-state systems [15]. Recently, it has become one of the most evolving technologies and the center of attraction for research to respond to various possible questions, which relate to the functioning of the devices that are based on this technique and also provide a variety of advance features in forthcoming industrial applications. Spintronic devices, due to the utilization of both carriers, spin and charge, seem favorable for a new variety of devices, such as polarized-light emitters, integrated-memory chips, microprocessor functions, and magnetic devices exhibiting gain ultra-low-power transistors. This advanced technology can make it possible to develop room-temperature ferromagnetic semiconductors, termed as dilute magnetic semiconductors (DMS), which exploit both the charge and the spin of electrons of the associated materials, simultaneously [16,17,18].

Moreover, CeO_2_-based nanomaterials have been demonstrated as potential active electrode materials, due to their potential technological applications in energy-storage devices. Among the energy-storage devices, supercapacitors are encouraging electrical energy-storage devices due to their higher power density, long cycle-life, and faster charging/discharging rate capacity than batteries [1,3,19] as well as their higher energy density compared to conventional electrical double-layer capacitors [20]. CeO_2_ is a low-cost material and exhibits excellent redox properties, due to the fast switching between its dual-oxidation states, i.e., Ce^3+^ and Ce^4+^. In addition, being a rare-earth element, the 4f electrons contribute to the electronic attributes of the material [21]. These properties make CeO_2_ a potential candidate for the preparation of the electrode materials to be utilized in supercapacitor applications. Mostly, there are two types of electrochemical capacitors based on the charge-storage mechanism, such as (1) electric double-layer capacitors (EDLCs) and (2) pseudocapacitors. EDLCs depend on non-Faradic charge separation, at the interface between an electrode and an electrolyte, and pseudocapacitors work based on electron transfer, which happens near the electrode/electrolyte. However, the existing literature, based on the supercapacitor applications of CeO_2_, is very limited. Moreover, the reported values of the specific capacitance of the electrodes prepared from CeO_2_ nanostructures are smaller. For instance, Prasanna et al. have reported a specific capacitance of ~134 F/g [22].

In the present work, we have investigated the structural, magnetic, and electrochemical properties of the chemically synthesized CeO_2_-undoped, Fe doped, and Fe plus Cu co-doped CeO_2_ nanoparticles. The nanoparticles were successfully prepared using the co-precipitation technique. The synthesized materials were characterized using various characterization techniques, viz. X-ray diffraction (XRD), scanning-electron microscopy (SEM), UV-visible spectroscopy, Raman spectroscopy, dc magnetization at room temperature, and electrochemical measurements.

## 2. Experimental

CeO_2_, Ce_0.98_Fe_0.02_O_2_, and Ce_0.78_Fe_0.02_Cu_0.20_O_2_ nanoparticles were prepared using the precursors Cerium (III) nitrate [Ce(NO_3_)_3_·6H_2_O; 432.22 g/mole; 99.99% purity], Ferric(III) nitrate [Fe(NO_3_)_3_·9H_2_O; 404 g/mole; 99.99% purity], and Copper(II) nitrate [Cu(NO_3_)_2_·3H_2_O; 241.592 g/mole; 99.99% purity], by the co-precipitation method. All the precursors were measured according to their stoichiometric ratios, to obtain the solution of 0.2 M, and then they were dissolved in 50 mL of DI water. The prepared solution was stirred for 3 h, using a magnetic stirrer at room temperature meanwhile, 30 min later, NH_4_OH solution was added drop by drop, until the pH reaches 9. Then, the prepared solution was centrifuged at 5000 rpm for 30 min. The particles obtained after centrifugation were washed three times with DI water and then twice with ethanol. Next, he particles obtained after washing were dried for 24 h at 60 °C. The gathered nanoparticles were ground for a few minutes to produce a fine powder. The samples were then calcined for 4 h at 500 °C and ground to obtain the desired nanoparticles.

The prepared nanoparticles were investigated using various characterization techniques. The XRD spectra of the prepared samples were recorded using the Philips X-pert X-ray diffractometer. The XRD pattern was measured with Cu Kα (λ = 1.5418 Å) radiations, in the range of 10° to 90°. The SEM micrographs were obtained by a field emission electron microscope (JSM-7500, JEOL, Tokyo, Japan). A UV-visible spectrophotometer was used to measure the optical absorption spectrum of the samples in the range of 200–800 nm. The Raman spectra was measured at Raman spectrometer (NRS-3100). The magnetic properties of the prepared samples were observed using the Quantum Design Physical Properties Measurement System, set up at room temperature. The electrochemical performance and specific capacitance of CeO_2_, Ce_0.98_F_0.02_O_2_, and Ce_0.78_Fe_0.02_Cu_0.20_O_2_ were studied using cyclic voltammetry (CV) at room temperature. CV measurements were demonstrated using an electrochemical analyzer (Corrtest-CS150). All electrochemical experiments were performed using a typical three-electrode experimental cell that had identical electrodes with respect to shape and size, made of the same active electrode materials. The electrodes were fabricated using materials (such as CeO_2_, Ce_0.98_F_0.02_O_2_, and Ce_0.78_Fe_0.02_Cu_0.20_O_2_), polyvinylidene fluoride (PVDF), and carbon black. The electrode materials were weighted in the ratio of 80:10:10 and mixed homogeneously, using n-methyl-2 pyrrolidinone (NMP) as a solvent to form a slurry. Finally, the slurry was coated onto the nickel foam and kept for drying at 80 °C in a hot air oven for 24 h. The CV measurements of the electrodes were performed in the range of 0.0 V to 0.6 V.

## 3. Results and Discussion

### 3.1. XRD Analysis

**Figure 1:**
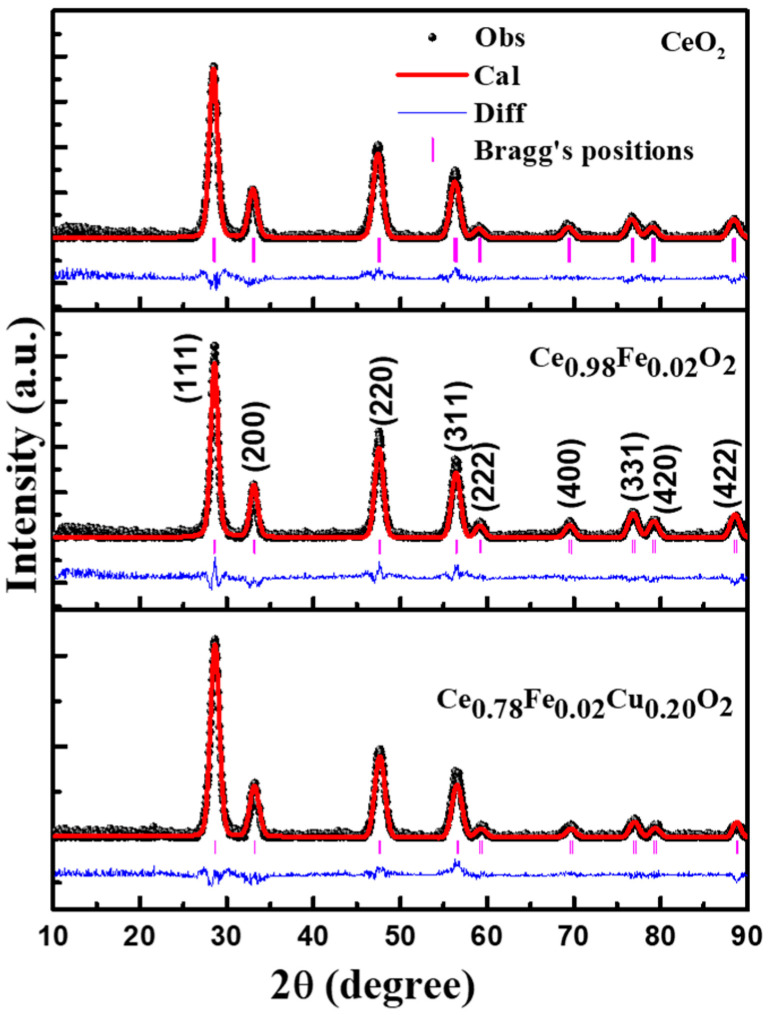
Rietveld refinement of pure CeO_2_, Ce_0.98_F_0.02_O_2_, and Ce_0.78_Fe_0.02_Cu_0.20_O_2_ nanoparticles.

**Figure 2:**
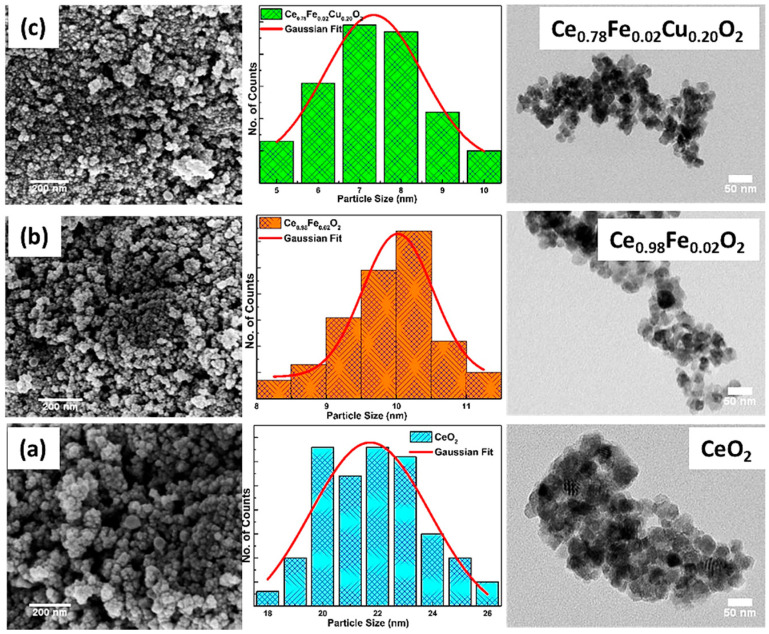
FE-SEM micrograph of (**a**) pure CeO_2_, (**b**) Ce_0.98_Fe_0.02_O_2_, and (**c**) Ce_0.78_Fe_0.02_Cu_0.20_O_2_ nanoparticles, along with the corresponding TEM images.

Figure 1 shows the typical XRD diffraction patterns of pure CeO_2_, Ce_0.98_Fe_0.02_O_2_, and Ce_0.78_Fe_0.02_Cu_0.20_O_2_ nanoparticles. It is observed that all the nanocrystalline samples indicate Bragg’s fundamental peaks, corresponding to the fluorite-type face-centered cubic structure, as per space group Fm-3m. According to this space group, Ce, Fe, and Cu atoms are located at the 4a position, surrounded by eight O, and then located at the 8b position. All the noticeable diffraction peaks in the XRD pattern corresponding to (111), (200), (220), (311), (222), (400), (331), (420), and (422) crystallographic planes clearly show the polycrystalline nature of the nanoparticles. The absence of any foreign peak corresponding to FeO, Fe_2_O_3_, Fe_3_O_4_, CuO, Cu_2_O, or any other complex surmises that Fe and Cu ions are successfully assimilated into the CeO_2_ matrix. From the investigation of the XRD spectra, it is detected that the location of the peaks initially shifted toward a lower 2*θ* value after Fe doping and then shifted towards a higher 2*θ* value as a consequence of Fe and Cu co-doping in CeO_2_. The shifting of peaks toward lower and higher angles signifies the lattice expansion and reduction, which is due to the successful doping of Fe and Cu ions into CeO_2_. Figure 2 displays the Rietveld refinement of the XRD patterns for all the samples. The Rietveld refinement was done using the Fullproof program, and the results are summarized in Table 1. The difference between the experimental and theoretically fitted curve has been shown by the blue line at the bottom, while the vertical pink lines indicate Bragg’s positions. Pure and doped CeO_2_ show diffraction peaks at 2*θ* = 28.37°, 32.89°, 47.31°, 56.17°, 58.88°, 69.24°, 78.93°, and 88.31°, for the respective crystallographic planes, and are in good agreement with JCPDS card no. 43-1002 [23]. The variation in the lattice parameters may be due to the difference in the ionic radii. The well-known Debye Scherer’s equation was used for the calculation of the average crystallite size of the samples, which can be written as:

$$D=\frac{K\lambda }{\beta \;Cos\;\theta }$$

where *D* is the average crystallite size of NPs, K is the shaping factor (≈0.9), λ is the wavelength of the incident radiation, *β* is the FWHM of the diffraction peaks, and *θ* is the semi-angle between the incident and diffracted rays. The crystallite size highlighted in Table 1 was found to decrease from 10.25 nm to 5.59 nm with Fe and Cu doping.

The spacing between two crystallographic planes, d, was calculated by the use of Bragg’s law, 2d Sin *θ* ꞊ nλ, where n is the integer value. With the help of “d”, the lattice parameter for each sample can be calculated with the formula

$$d=\frac{a}{\sqrt{h^{2}+k^{2}+l^{2}}}$$

The lattice parameters are summarized in Table 1. It is identified that the lattice parameter for pure CeO_2_ was 5.442 Ǻ, which increases to 5.447 Ǻ and then, finally, decreases to 5.434 Ǻ, for 2% Fe and 2% Fe plus 20% Cu doping, respectively. The incorporation of Fe and Cu ions doping in CeO_2_ nanoparticles may generate localized strain in all the samples. Therefore, we have determined the lattice strain using Wilson’s formula  β=4ε tanθ, where *β* is FWHM, *ε* is lattice strain, and *θ* is Bragg’s angle. In fact, the particle size and the lattice strain have an independent effect on the FWHM; therefore, they are independent of each other. Table 1 summarized the strain of all the samples calculated using the W-H plot. As can be seen from Table 1, the strain is found to increase with Fe and Cu doping in CeO_2_. It is noticed that all the strain values are positive, and these positive values of strain are related to the tensile strain in Fe and Cu codoped CeO_2_ nanoparticles. Some theoretical reports published in the literature indicate that the tensile strain indorses in the development of oxygen vacancies more than compressive strain. Therefore, an increase in the tensile strain in the Fe and Cu codoped CeO_2_ samples can be directly related to an increase in oxygen vacancies. Meanwhile, in the case of tensile strain, it is observed that the bandwidth of O 2p orbital decreases, which also results in a decrease in the overlapping between O 2p orbitals and Ce 5d orbitals as well as Ce 4f orbitals. This decrease in the overlapping of orbitals leads to a weaker Ce-O bond and is accountable for the formation of oxygen vacancies.

### 3.2. Morphological Analysis

The micrographs obtained using transmission-electron microscopy (TEM) and scanning-electron microscopy (SEM) are displayed in Figure 2a–c. The micrographs reveal a spherical-shape morphology with particle sizes in the nm range. The micrographs clearly reveal that the particle size reduces after doping. The average particle is obtained to be ~21.0 nm, 10.0 nm, and 7.0 nm for CeO_2_, Fe doped CeO_2,_ and Fe plus Cu co-doped CeO_2_ nanoparticles, respectively. A similar decreasing trend of particle size is also observed in the TEM micrographs. The variation of average particle sizes is in accordance with the average crystallite sizes obtained from XRD analysis.

### 3.3. UV-Visible Spectroscopy

**Figure 3:**
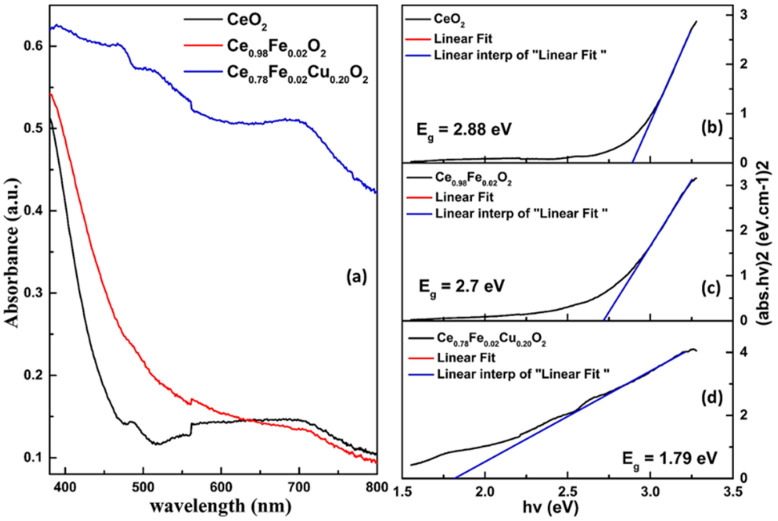
(**a**) UV–Vis absorption spectra of pure CeO_2_, Ce_0.98_Fe_0.02_O_2_, and Ce_0.78_Fe_0.02_Cu_0.20_O_2_ nanoparticles. Tauc’s plot of (**b**) pure CeO_2_ nanoparticles, (**c**) Ce_0.98_Fe_0.02_O_2_, and (**d**) Ce_0.78_Fe_0.02_Cu_0.20_O_2_.

The optical properties of pure CeO_2_, Ce_0.98_Fe_0.02_O_2_, and Ce_0.78_Fe_0.02_Cu_0.20_O_2_ nanoparticles were studied using UV-Visible spectroscopy. The absorption spectra, due to electronic excitation from the valence band (VB) to the conduction band (CB) in the UV ranges, reflects the width of the bandgap energy. The variation in the content concentration of dopant ions modifies the intensity of absorbance and alters the position of the peak, which indicates the successful incorporation of dopant ions in the lattice matrix, which causes a modification in the electronic structure of CB and VB, i.e., CB is mainly formed from O-2p and Ce-4f (Cu/Fe-3d) states near the Fermi level. Hence, it is expected that the change in dopant content is associated with a change in electronic structure and bandgap energy, due to the mismatch between the ionic radii of the dopant and the host, which enhances the formation of O vacancies and simultaneously changes the electronic bands, defect sites, and impurity centers. Further, the optical-band-gap energy is equivalent to the required photon energy for exiting the electron from VB to CB. The optical-bandgap energy has been calculated using the absorption spectrum, with the help of the Tauc’s equation, which can be written as:

(αhν)^1/n^ = A (hν−E_g_)

where h is Planck’s constant, ν is the photon’s frequency, α is the absorption coefficient, E_g_ is the bandgap, and A is the proportionality constant. For the calculation of the direct bandgap of the samples, the value of n is taken as 1/2, as shown in Figure 3. The energy-bandgap of pure CeO_2_, Ce_0.98_Fe_0.02_O_2_, and Ce_0.78_Fe_0.02_Cu_20_O_2_ is determined to be 2.88 eV, 2.7 eV, and 1.79 eV respectively. A decrease in the optical-energy bandgap has been noticed with the doping and co-doping. This is because the oxygen vacancies give rise to the defect states in the bandgap region. Besides, the d-states of the dopant ions split, due to spin-orbit-coupling effects into the e_g_ and t_2g_ states, which increase the density of states in the valence band of the material. Thus, the minimum bandgap has been observed to be 1.79 eV in the 20% Cu co-doped with 2% Fe doped CeO_2_.

### 3.4. Raman Spectroscopy

**Figure 4:**
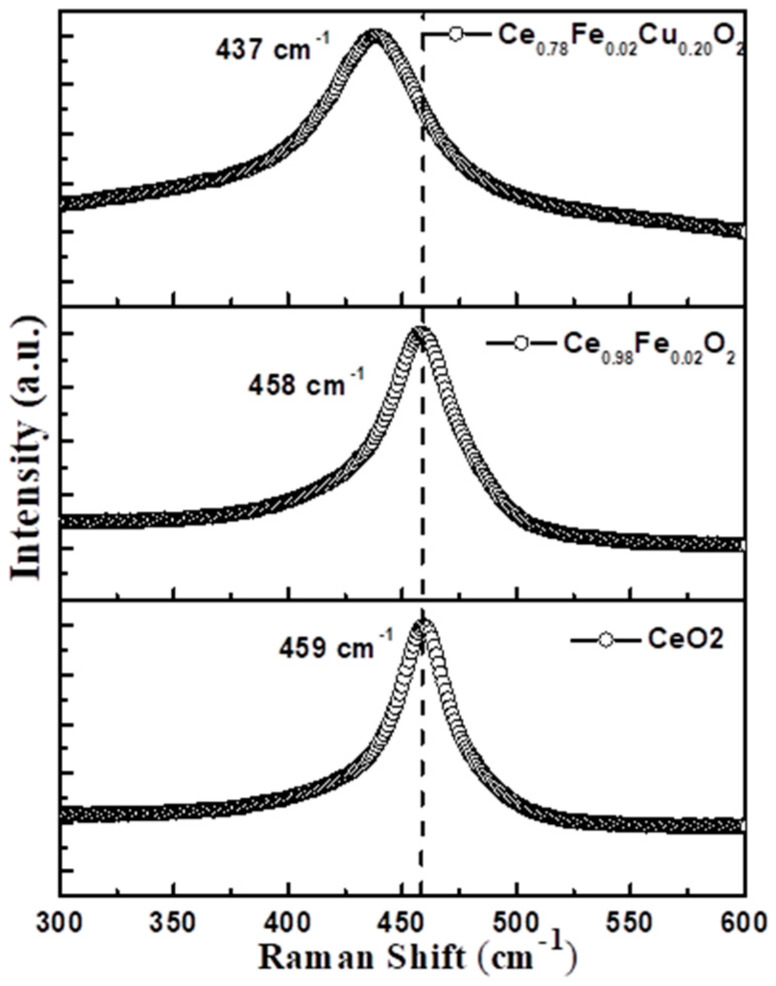
Raman spectra of pure CeO_2_, Ce_0.98_Fe_0.02_O_2_, and Ce_0.78_Fe_0.02_Cu_0.20_O_2_ nanoparticles.

Raman spectroscopy is used to investigate the stoichiometry of the Ce-O sublattice by analyzing the vibrational modes. The Raman spectra of the samples are displayed in Figure 4, which exhibit single bands corresponding to the F_2g_ Raman active modes of CeO_2_ cubic structure. Generally, the Raman active mode of CeO_2_ is observed at 460 cm^−1^. In the present case, the Raman active mode for undoped CeO_2_ is observed at 459 cm^−1^. The mode shifts towards lower Raman frequency after doping, observed at 458 cm^−1^ for Ce_0.98_Fe_0.02_O_2_ and 437 cm^−1^ for Ce_0.78_Fe_0.02_Cu_0.20_O_2_. The shift in the F_2g_ modes may be attributed to the presence of the defect states. The defect states in the CeO_2_ are likely to be the oxygen vacancies because of the rapidly changing behavior of the oxidation states of Ce (3+/4+).

### 3.5. Magnetic Properties

**Figure 5:**
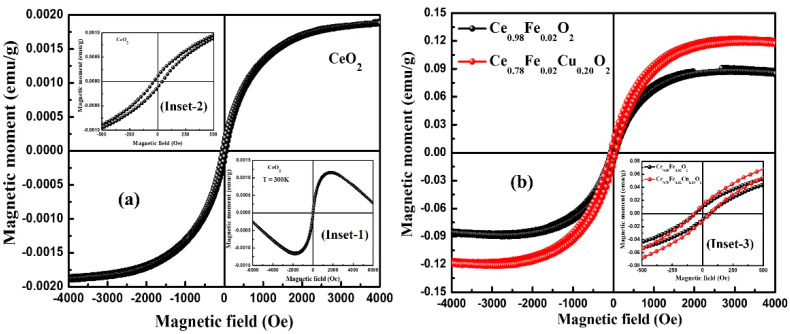
(**a**,**b**) M-H plots of CeO_2_, Ce_0.98_Fe_0.02_O_2_, and Ce_0.78_Fe_0.02_Cu_0.20_O_2_ nanoparticles. Inset-1 shows the M-H curve of pure CeO_2_ nanoparticles along with diamagnetic contribution in the field range of ±6000 Oe. Inset-2 represents the M-H curve of CeO_2_ nanoparticles in low-field range (±500 Oe). Inset-3 represents the M-H curve of Ce_0.98_Fe_0.02_O_2_ and Ce_0.78_Fe_0.02_Cu_0.20_O_2_ nanoparticles in low-field range (±500 Oe).

Figure 5a,b display the magnetization (M) versus magnetic field (H) curve of pure CeO_2_, Ce_0.98_FeO_2_, and Ce_0.78_Fe_0.02_Cu_0.20_O_2_ nanoparticles recorded at room temperature (RT). The M-H curve of pure CeO_2_, after subtracting the diamagnetic contribution, is shown in Figure 5a. Inset-1 in Figure 5a highlights a magnified view of the M-H hysteresis curve of CeO_2_ nanoparticles along with the diamagnetic contribution. Inset-2 represents the M-H curve of undoped CeO_2_ in the low-field (±500 Oe) region. Figure 5b shows the M-H curve of Ce_0.98_Fe_0.02_O_2_ and Ce_0.78_Fe_0.02_Cu_0.20_O_2_ nanoparticles. Inset-3 denotes a magnified view of the M-H curve in the low-field (±500 Oe) region. It is observed that a pure CeO_2_ nanoparticle (see Figure 5a) also displays weak ferromagnetic (FM) ordering at room temperature (RT), with small values of parameters such as the coercive field (H_C_ = 32 Oe), remanence magnetization (M_R_ = 1.1 × 10^−4^ emu/g), and saturation magnetization (M_S_ = 1.9 × 10^−3^ emu/g). Here, it is worth to mention that bulk CeO_2_ displays nonmagnetic behavior at room temperature, but a pure CeO_2_ nanoparticle demonstrates weak ferromagnetism, which might be associated with oxygen vacancies [24,25]. Moreover, various magnetic parameters calculated from the M-H curve are displayed in Table 2. It is found that the saturation magnetization of Ce_0.98_Fe_0.02_O_2_ and Ce_0.78_Fe_0.02_Cu_0.20_O_2_ nanoparticles increases from 9.0 × 10^−2^ emu/g to 12.2 × 10^−2^ emu/g, respectively. It is noticeable that the saturation-magnetization value increases significantly on doping and co-doping CeO_2_ with Fe and Cu, respectively. The remanent magnetization (M_R_) for Ce_0.98_Fe_0.02_O_2_ and Ce_0.78_Fe_0.02_Cu_0.20_O_2_ nanoparticles, determined using the M-H curve, was found to increase from 9.7 × 10^−3^ emu/g to 13.0 × 10^−3^ emu/g, and the corresponding coercive field (H_C_) was 60.0 Oe and 68.0 Oe, respectively. The observed change in M_S_ indicates the increment in ferromagnetism in the host material. The possible cause of the enhancement of the ferromagnetic ordering is the formation of oxygen vacancies, which originate when the smaller size cations substitute for the host cation. The origin of FM in CeO_2_ might be explained using the F-center-exchange-coupling (FCE) mechanism. In the last few decades, various models such as carrier-mediated exchange, donor-impurity-band exchange, the bound-magnetic-polaron model (BMP), etc. in DMSs, have been used to explain the FM behavior of the dilute magnetic semiconductors [6,26,27,28,29,30]. Lately, Coey et al. [28] proposed an FCE mechanism for explaining the origin of high-temperature FM in insulating oxides. The FM ordering observed in Ce_0.98_Fe_0.02_O_2_ and Ce_0.78_Fe_0.02_Cu_0.20_O_2_ nanoparticles might be explained using the FCE mechanism. As per the FCE model, magnetic ion and oxygen vacancy (Ov) are the two necessities for FM ordering. According to this mechanism, Ov as surface defects plays a significant part in the origin of FM. The electrons trapped in oxygen vacancies constitute groups with two transition-metal ions (i.e., TM–Ov–TM, wherein Ov represents oxygen vacancy). As a result of this, the electron confined in an oxygen vacancy occupies an orbital that overlaps with the d shell of the neighboring TM ions, which establishes the spin orientations of neighboring TM ions. In agreement with Hund’s rule and Pauli’s exclusion principle, ferromagnetic ordering is established because the spin of the trapped electrons would be in the direction parallel to that of the two neighboring TM ions. The RTFM observed in pure and Fe plus Cu co-doped CeO_2_ nanoparticles indicates that the material might be suitable for spintronic applications.

### 3.6. Electrochemical Properties

**Figure 6:**
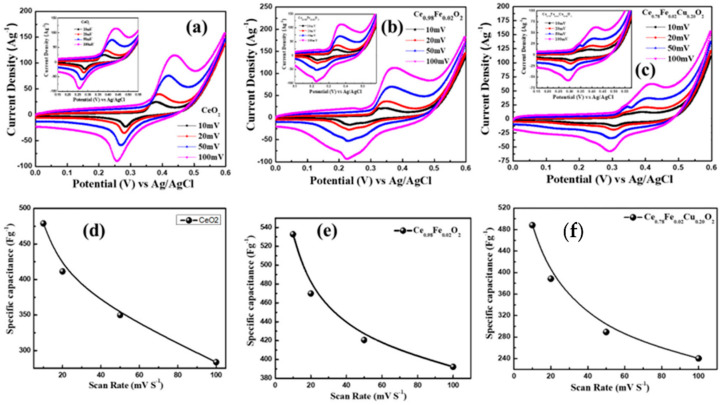
(**a**–**c**) CV plots of CeO_2_, Ce_0.98_Fe_0.02_O_2_, and Ce_0.78_Fe_0.02_Cu_0.20_O_2_ nanoparticles with different scan rates. (**d**–**f**) Variation in specific capacitance of CeO_2_, Ce_0.98_Fe_0.02_O_2_, and Ce_0.78_Fe_0.02_Cu_0.20_O_2_ nanoparticle electrodes with different scan rates.

The electrochemical properties of the electrodes of CeO_2_, Ce_0.98_F_0.02_O_2_, and Ce_0.78_Fe_0.02_Cu_0.20_O_2_ nanoparticles were evaluated using cyclic-voltammetry (CV) measurements in the 2M KOH electrolyte. Figure 6a–c represent the CV curve of CeO_2_, Ce_0.98_F_0.02_O_2_, and Ce_0.78_Fe_0.02_Cu_0.20_O_2_ electrodes, recorded in the range of 0.0 V and 0.6 V, at different potential scan rates of 10–100 mV s^−1^. It is evident from Figure 6a–c that the shape of the CV curves of all the materials are similar, and the current response was found to increase with an increase in the scan rate. One can notice from Figure 6a–c that the height of the peaks shifted to the higher potential in the CV curve, when the scan rate is increased from 10 mV s^−1^ to 100 mV s^−1^. Figure 6d–f highlight the specific capacitance of CeO_2_, Ce_0.98_F_0.02_O_2_, and Ce_0.78_Fe_0.02_Cu_0.20_O_2_ electrodes, calculated at different scan rates of 10–100 mV s^−1^. It is observed that all the electrodes exhibit the highest value of the specific capacitance at the scan rate of 10 mV s^−1^, which starts decreasing with an increase in the scan rate.

**Figure 7:**
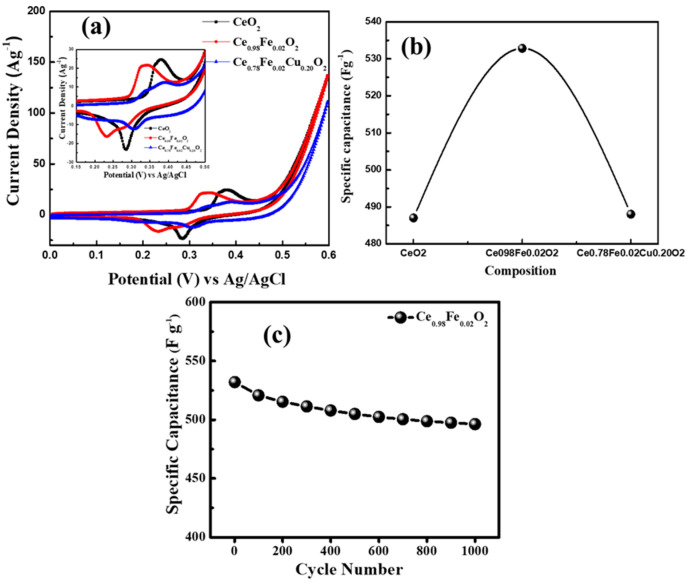
(**a**) CV plots of CeO_2_, Ce_0.98_Fe_0.02_O_2_, and Ce_0.78_Fe_0.02_Cu_0.20_O_2_ nanoparticles at scan rate of 10mV S^−1^. (**b**) Variation of specific capacitance of CeO_2_, Ce_0.98_Fe_0.02_O_2_, and Ce_0.78_Fe_0.02_Cu_0.20_O_2_ nanoparticles at scan rate of 10mV S^−1^. (**c**) Cyclic performance of Ce_0.98_Fe_0.02_O_2_ electrode for 1000 cycles.

Figure 7a show the comparison of the CV plots of CeO_2_, Ce_0.98_F_0.02_O_2_, and Ce_0.78_Fe_0.02_Cu_0.20_O_2_ nanoparticles at the scan rate of 10 mV s^−1^. It can be seen from Figure 7a that the Ce_0.98_F_0.02_O_2_ electrode exhibits the maximum area under the curve, which reveals the highest capacitive reaction compared to the other electrodes. The values of the specific capacitance were calculated using the relation $C=\frac{1}{2\mathrm{mVk}}\int_{V^{-}}^{V^{+}}I\left(V\right)\mathrm{dV}$, where m (g) denotes the mass of the active material and k (V s^−1^) is scan rate, C (F g^−1^) represents the specific capacitance, and I (A) symbolizes the discharge current [31]. The values of the specific capacitance of CeO_2_, Ce_0.98_F_0.02_O_2_, and Ce_0.78_Fe_0.02_Cu_0.20_O_2_ electrodes (see Figure 7b), determined using the CV curve at the scan rate of 10 mV s^−1^, were 487.0 F g^−1^, 532.8 F g^−1^, and 488.0 F g^−1^, respectively. Moreover, it is found that the specific capacitance of the Ce_0.98_F_0.02_O_2_ electrode decreases (see Figure 6e) from ~532.8 F g^−1^ to ~392.17 F g^−1^ when increasing the scan rate from 10 mV s^−1^ to 100 mV s^−1^. It is important to mention here that the CV curves exhibit a rectangular shape characteristic, which shows the ideal pseudo-capacitive behavior of the electrodes. The electrochemical stability of the Ce_0.98_F_0.02_O_2_ electrode was measured by repeating the CV measurements for 1000 cycles. Figure 7c represents the variation in the specific capacitance after 1000 cycles, which demonstrated remarkable cyclic stability with capacitance retention of ~94% for 1000 cycles. In the present-studied samples, it is observed that the Ce_0.98_F_0.02_O_2_-based electrode showed superior performance than the other CeO_2_-based electrodes that were reported in the literature (see Table 3). Padmanathan et al. [32] reported a high capacitance of 644 F g^−1^ at 0.5 A g^−1^ and 400 F g^−1^ at a high-current density of 20 A g^−1^. In another report, the specific capacitance of 162.47 F g^−1^, 149.03 F g^−1^, and 87.73 F g^−1^ was observed for CeO_2_ nanorods, CeO_2_ nanotubes, and CeO_2_ nanoparticlesm respectively [3]. Aravinda et al. also studied the supercapacitor behavior of nano CeO_2_/activated-carbon composite and they found the specific capacitance of ~162 F g^−1^ [33]. Furthermore, Deng et al. [19] fabricated CeO_2_ and CeO_2_ nanoparticles decorated with graphene oxide and reported an improvement in the specific capacitance from 81.18 F g^−1^ (CeO_2_) to 382.94 F g^−1^ (CeO_2_/GO (1:4)). Therefore, it is evident from the literature that the Ce_0.98_F_0.02_O_2_ electrode showed the highest value of the specific capacitance at the current density of 23 A g^−1^, which might be a favorable electrode material for supercapacitor applications.

## 4. Conclusions

The co-precipitation method was used for the successful preparation of CeO_2_, Ce_0.98_Fe_0.02_O_2_, and Ce_0.78_Fe_0.02_Cu_0.20_O_2_ nanoparticles. X-ray-diffraction analysis concludes that all the samples show a single-phase face-centered cubic (fluorite) structure. The absence of any foreign peak in the XRD pattern reveals that Fe and Cu ions are successfully replacing Ce ions in the CeO_2_ host matrix. The crystallite size, determined from the XRD pattern using Scherrer’s formula, was found to be in the range of 5.59–10.25 nm. Raman spectra revealed F_2g_ active modes corresponding to the Ce-O_8_ vibrational units of CeO_2_. The UV-Vis spectra were found to exhibit a strong broadband between 200–800 nm. The energy bandgaps, calculated using Tauc’s plots, were found to decrease from 2.88 eV to 1.79 eV with an increase in the dopant concentration, showing a red shift. The dc-magnetization measurements demonstrate that saturation magnetization increases with an increase in the doping ions. The remnant-magnetization values of the samples indicate that the nanoparticles possess weak ferromagnetic behavior at room temperature. The highest value of saturation magnetization (12.2 × 10^−2^ emu/g) has been found for Ce_0.78_Fe_0.02_Cu_0.20_O_2._ Electrochemical studies demonstrated that the Ce_0.98_F_0.02_O_2_ electrode is a better electrode for supercapacitors, with a specific capacitance of 532 F g^−1^ measured at a 10 mV s^−1^ scan rate, along with an excellent capacitance retention of ~94% for 1000 cycles.

## Figures and Tables

**Table 1:** The values of various parameters obtained, such as crystallite size (D), FWHM (d), lattice strain through XRD, and bandgap energy (Eg) from UV-vis analysis of pure CeO_2_, doped CeO_2_, and co-doped CeO_2_.

Sample	D (nm)	d_(111)_ (nm)	a (Å)	Strain ×10^−2^	E_g_ (eV)
CeO_2_	10.25	0.3142	5.442	1.02	2.88
Ce_0.98_Fe_0.02_O_2_	8.57	0.3145	5.447	1.21	2.7
Ce_0.78_Fe_0.02_Cu_0.20_O_2_	5.59	0.3137	5.434	2.22	1.79

**Table 2:** The values of various parameters, such as coercive field (H_C_), remanence magnetization (M_R_), and saturation of magnetization (M_S_) obtained through dc-magnetization-measurements analysis of pure CeO_2_, doped CeO_2_, and co-doped CeO_2_.

Sample	M_R_ (emu/g)	H_C_ (Oe)	M_S_ (emu/g)
CeO_2_	1.1 × 10^−4^	38.0	1.9 × 10^−3^
Ce_0.98_Fe_0.02_O_2_	9.7 × 10^−3^	60.0	9.0 × 10^−2^
Ce_0.78_Fe_0.02_Cu_0.20_O_2_	13 × 10^−3^	68.0	12.2 × 10^−2^

**Table 3:** A comparative study of specific capacitance between current work and earlier reports.

Electrode Materials	Specific Capacitance (F g^−1^)	Ref.
CeO_2_	81.18	[19]
CeO_2_/GO (1:1)	45.29	
CeO_2_/GO (1:2)	82.35	
CeO_2_/GO (1:4)	382.94	
CeO_2_/GO (1:5)	18.37	
CeO_2_ nanorods	162.47	[3]
CeO_2_ nanocubes	149.03	
CeO_2_ nanoparticle	87.73	
CeO_2_	400	[32]
Nano CeO_2_/activated carbon	162	[33]
CeO_2_	487	This work
Ce_0.98_Fe_0.02_O_2_	532	
Ce_0.78_Fe_0.02_Cu_0.20_O_2_	488	

## Data Availability

Not applicable.
